# Supplementary material for: MetaRibo-Seq measures translation in microbiomes
Source: Nat Commun. 2020 Jun 29;11:3268. doi: 10.1038/s41467-020-17081-z (PMC7324362; doi:10.1038/s41467-020-17081-z)
Supplement: Supplementary file 10 — Supplementary Data 7 [file 41467_2020_17081_MOESM10_ESM.zip › File2/Confidence_VeryHigh_Taxonomy/224351_out.krona.html]

Javascript must be enabled to view this page.

members
magnitude
magnitudeUnassigned
count
unassigned
taxon
rank

224351\_out

15

15
superkingdom
2

phylum
32066
15

15
class
203490

203491
order
15

203492
family
1

848
genus
1

860

SRS143604\_contig\_number\_contig-100\_9227.51486
species
1

1129771
family
14

14
32067
genus

157687
species

SRS015434\_contig\_number\_20138SRS016456\_contig\_number\_contig-100\_5532.5533SRS022143\_contig\_number\_31399SRS049253\_contig\_number\_15737SRS098498\_contig\_number\_45072SRS149486\_contig\_number\_21340SRS893322\_contig\_number\_contig-100\_5422.5423
7

2
109328

SRS018739\_contig\_number\_contig-100\_819.115306.115306SRS045715\_contig\_number\_48393
species

3

SRS023352\_contig\_number\_contig-100\_8571.189955.189955SRS146738\_contig\_number\_contig-100\_5844.106160SRS147178\_contig\_number\_contig-100\_1963.148183
species
1785996


SRS075347\_contig\_number\_contig-100\_12697.75648SRS1055084\_contig\_number\_contig-100\_6043.139756
species
671213
2
